# Enhancing fragment-based protein structure prediction by customising fragment cardinality according to local secondary structure

**DOI:** 10.1186/s12859-020-3491-0

**Published:** 2020-05-01

**Authors:** Jad Abbass, Jean-Christophe Nebel

**Affiliations:** 1 Faculty of Science, Engineering and Computing, Kingston University, London, KT1 2EE UK; 2 Department of Computer Science, Lebanese International University, Bekaa, Lebanon

**Keywords:** Fragment-based protein structure prediction, Rosetta, Protein secondary structure

## Abstract

**Background:**

Whenever suitable template structures are not available, usage of fragment-based protein structure prediction becomes the only practical alternative as pure ab initio techniques require massive computational resources even for very small proteins. However, inaccuracy of their energy functions and their stochastic nature imposes generation of a large number of decoys to explore adequately the solution space, limiting their usage to small proteins. Taking advantage of the uneven complexity of the sequence-structure relationship of short fragments, we adjusted the fragment insertion process by customising the number of available fragment templates according to the expected complexity of the predicted local secondary structure. Whereas the number of fragments is kept to its default value for coil regions, important and dramatic reductions are proposed for beta sheet and alpha helical regions, respectively.

**Results:**

The evaluation of our fragment selection approach was conducted using an enhanced version of the popular Rosetta fragment-based protein structure prediction tool. It was modified so that the number of fragment candidates used in Rosetta could be adjusted based on the local secondary structure. Compared to Rosetta’s standard predictions, our strategy delivered improved first models, + 24% and + 6% in terms of GDT, when using 2000 and 20,000 decoys, respectively, while reducing significantly the number of fragment candidates. Furthermore, our enhanced version of Rosetta is able to deliver with 2000 decoys a performance equivalent to that produced by standard Rosetta while using 20,000 decoys. We hypothesise that, as the fragment insertion process focuses on the most challenging regions, such as coils, fewer decoys are needed to explore satisfactorily conformation spaces.

**Conclusions:**

Taking advantage of the high accuracy of sequence-based secondary structure predictions, we showed the value of that information to customise the number of candidates used during the fragment insertion process of fragment-based protein structure prediction. Experimentations conducted using standard Rosetta showed that, when using the recommended number of decoys, i.e. 20,000, our strategy produces better results. Alternatively, similar results can be achieved using only 2000 decoys. Consequently, we recommend the adoption of this strategy to either improve significantly model quality or reduce processing times by a factor 10.

## Background

Generally, a protein is not functional, and may even be harmful, unless it folds into its unique shape. Although a protein’s possible conformation space is huge, in nature, the folding process often occurs on a timescale of microseconds, which has led to the formulation of the so-called Levinthal’s Paradox that still remains unsolved [1–4]. Investigation of folding pathways has started to reveal important clues helping computational biologists to understand the actual trajectory that a protein follows towards its native structure [5, 6]. For globular proteins, the hydrophobic effect has been identified as an essential factor: hydrophobic amino acids tend to aggregate in the centre of the structure to avoid the surrounding water molecules, whilst the hydrophilic ones prefer to stay in contact with the external aqueous environment. However, how a protein reaches its final 3D structure that is highly related to its biological function remains a mystery even if such conformation is believed to usually display the lowest free energy [7].

So far, the only trusted and formal ways to determine a protein’s 3D structures are via experimental techniques, namely, X-Ray crystallography, Nuclear Magnetic Resonance (NMR) and Electron Microscopy (EM). Only structures resolved by those means can be deposited in the single worldwide repository of large biological molecules: the Protein Data Bank (PDB) [8]. Since those experimental techniques are very expensive, time consuming and often inconclusive, there is a strong incentive in generating such knowledge via computational means. Indeed, protein structure prediction (PSP), i.e. predicting computationally the 3D structure of proteins from their sequences of Amino Acids (AAs), has been referred to as the “holy grail of computational biochemistry” [9]. Unfortunately, this has also been claimed to be one of the most complicated optimisation problems computer scientists have ever faced [3].

Since 1994, the field of PSP has been monitored, quantitatively evaluated and stimulated by the biennial CASP (Critical Assessment of protein Structure Prediction) events. These community-wide experiments have grown significantly from a set of 33 targets which attracted around 100 models (CASP1, 1994) to a set of 82 targets which led to the submission of more than 55,000 models (CASP13, 2018). Every two years, a set of protein sequences are released gradually across a couple of months during which research groups from around the world attempt to predict their 3D structures by submitting putative models (up to 5 per target). Those protein sequences come from joint experimental laboratories where determination of their native structures is planned to be conducted in vitro. Once a target’s submission deadline has passed, a thorough evaluation is performed on the submitted models, if experiment to determine its native structure has been successfully carried out and no early release of the tertiary structure has taken place. In the first 6 rounds – that is, till CASP6 – targets were classified into three categories: ‘comparative modelling’, ‘fold recognition’ and ‘ab initio’ (or ‘new fold’). Since then, targets have been annotated according to only two classes: ‘template-based modelling’ (TBM) and ‘free modelling’ (FM). Whereas the TBM category comprises ‘easy targets’ for which structures of homologous proteins have already been deposited in the PDB, FM targets represent the greatest challenge in the competition since only research groups that rely on ab initio methods can contribute. Due to the complexity of that task, even minor improvements in FM prediction accuracy amongst competing groups are considered worthwhile.

Christian Anfinsen – one of the pioneers in the study of relationships between structure and function in proteins – has formulated two significant theories: i) the native structure is the one that has the lowest free energy value [7], and ii) protein folding is a pure physical process, i.e. the tertiary structure can be solely determined by the sequence of amino acids [10]. The above two principles represent the bases for ab initio protein structure prediction. From the first theory’s perspective, PSP is an optimisation problem where an energy function can play the role of heuristic while attempting to reach the global minimum energy within the search space. Anfinsen’s second theory has paved the way to computationally estimate the interactions that take place between atoms without taking into consideration any external effect. Whereas performing protein folding simulations conforming to Newton’s second law may appear as an attractive approach, it is only practical when applied to very small targets while using state-of-the-art supercomputers and/or grid computing [11]. Indeed, even for short protein sequences, the search space is enormous and is computationally intractable (an NP-hard problem) [2].

In principle, ab initio approaches do not rely on previous known structures. They are based on thermodynamic rules expressing interactions amongst atoms and energy functions and, thus, the most stable structure can be found by determining the minimum energy configuration through a Force Field (FF) energy model. Such model aims at evaluating structures using an energy-scoring function that usually quantifies chemical interactions and physical forces that occur within the conformation. Ab initio approaches rely on searching and sampling techniques such as molecular dynamics [12], Monte Carlo [9, 13], simulated annealing [14] and genetic algorithms [15]. However, not only do all those techniques suffer from the trade-offs [16, 17] associated with explore-and-exploit algorithms [18, 19], but also, as the search space is enormous, they tend to converge towards local minima. Despite this limitation, ab initio methods are of great interest since they are the only ones that can, in principle, derive the native structure of any protein. Moreover, they may give insights into folding mechanisms and pathways that are other great challenges of molecular biology [20]. While the ‘classical’ definition of ab initio imposes the amino acid sequence to be the sole source of data, some methods started to use short structural fragments, i.e. a set of amino acids rather than a single amino acid [21, 22], creating the ‘coarse grained’ or ‘fragment-based’ ab initio sub-category. The launch of approaches that involve longer fragments such as ROBETTA [22], I-TASSER [23] and QUARK [24] has further widened the gap between those two types of ab initio modelling approaches.

Despite enormous advances in PSP during the past two decades, all methods suffer from inconsistency: although they may be successful at predicting some particular targets, they fail for others [25, 26]. In addition, they tend to be unable to deal with large- and medium-size proteins (i.e. with 300+ residues): only template-based models associated with very high sequence identity display accuracies comparable to experimental techniques’ [26].

Although, for almost two decades, Rosetta and other fragment-based PSP approaches, such as I-TASSER and QUARK, dominated FM target predictions, the latest round of CASP - CASP13 – dramatically changed that situation. Indeed, the exploitation of deep learning techniques led to significant improvement of inter-residues distance predictions that are as restraints for tertiary structure prediction [27]. Consequently, CASP13 revealed ‘unprecedented success’ in overall FM results [28]: the GDT averages of the best models submitted for FM targets in CASP12 and CASP13 increased from 52.9 to 65.7 respectively. Among approaches relying on deep learning, AlphaFold by Google’s DeepMind [29, 30] predicted many targets with outstanding and unprecedented accuracies [31] and outperformed significantly all its competitors with a cumulative z-score of 94.7. The second best group -“Zhang” -, which takes advantage of two fragment-based PSP approaches I-TASSER and QUARK, only achieved 67.2. Although such a domination of AlphaFold at its first participation shocked the CASP community, there is no doubt that it will have learned and adapted to offer competitive PSPs at the coming CASP14 (2020). While some groups will follow a pure deep learning approach as AlphaFold did, others will continue to enhance their fragment-based PSP approaches by, among other things, integrating deep learning-based predictions. The work presented in this manuscript should prove particularly relevant to the latter.

After a review of fragment-based PSP approaches, resampling techniques and a description of the Rosetta tool, we propose to improve fragment-based modelling by customising fragment selection according to the protein of interest. More specifically, the number of available fragments used at each position of the model being constructed is chosen depending on the secondary structure associated to the position of interest. Using a dataset of average and high complexity targets, this new scheme is evaluated in terms of model quality and processing requirements.

### Fragment-based protein structure prediction

These methods, first, search in the PDB for known structure fragments that match sub-sequences of the protein of interest. Once candidate fragments have been selected, compact structures can be formed by randomly assembling fragments using stochastic techniques such as simulated annealing. Then, the fitness of each conformation is evaluated and the most promising ones are optimized: while physics-based methods rely on explicitly optimising free-energy, fragment-based approaches perform a similar task by using scoring functions that are loosely related to energy functions. Besides, it has been shown that including a similarity measure between the secondary structure of a candidate fragment and the corresponding predicted one in the target improves scoring functions [29].

Fragment-based approaches are much less computationally expensive than ‘classical’ ab initio PSP ones for three main reasons: (1) since they are ‘coarse grained’ - the computation unit is a set of amino acids instead of a single one -, the conformational search space is decreased dramatically, (2) Monte Carlo simulations are used instead of Molecular Dynamics, and (3) since used fragments are already of low-energy, local interactions do not need to be calculated within the fragments that are introduced in the structure. In order to cope with the large search space, most fragment-based PSP computational tools rely on generating thousands of candidate structures, known as decoys, where each of them represents, in principle, a different search trajectory. Typically, the decoy(s) with the lowest energy score is (are) then considered as the best prediction(s). Success of PSP using fragment assembly relies on three fundamentals: energy function accuracy, search method efficiency and quality of the fragment library [30]. Indeed, as a key principle behind fragment-based PSP is that any structure can be built from the concatenation of fragments from protein structures available in the PDB [31], an ideal fragment library should be able to build any FM target.

Weakness in any of them yields wrong search trajectories, thus, inadequate quality of decoys. The majority of fragment packing methods use a coarse-grained atomic representation during the sampling phase so that the smoothness of the search space helps avoiding local minima. Full-atom representation is then obtained gradually during optimisation and refinement phases, mostly using knowledge-based ideal values. Nevertheless, fragment-based methods continue to fail reaching reasonable accuracy for many CASP’s targets, which has paved the way for further investigations, comparative studies, improvements, amendments and tuning [30, 32–45].

Among the many fragment-based PSP packages that have been developed [46], FRAGFOLD [47], I-TASSER [48], QUARK [24] and Rosetta [49] have proved the most successful. Developed by Jones in 1996 [50], FRAGFOLD may be considered to be the first fragment-based method. The performance of this innovative approach in CASP2 (1996) proved particularly promising and paved the way for the development of similar methods. Its main contribution was the usage of two types of fragments: super-secondary structural motifs (variable length of 9 to 31 residues) which were shown to be parts of the polypeptides that form early and remain stable during the folding process, and miscellaneous fragments extracted from high-resolution proteins (fixed length of 9-mers). FRAGFOLD was continuously improved until 2005 [51–53]: while in CASP6, it achieved reasonable accuracy for 4 out of 8 targets in the FM category (called ‘New Fold’ then), it delivered excellent results in CASP9, where it was overall ranked 24th out of 174 in terms of the first model.

Following the creation of TASSER (Threading/ASSEmbly/Refinement) in 2004 [54], its Iterative version (I-TASSER [55]) proved the most successful. It is a hierarchical approach that combines ab initio modelling and threading. Since the length of the fragments chosen from threading has no upper limit (greater than or equal to 5), this method is suitable for both FM and TBM targets. I-TASSER initially generates low resolution conformations that are then refined. More specifically, structure prediction relies on three main stages [56]. First, sequence profile and predicted secondary structure are used for threading through a representative set of the PDB. The most highly-ranked template hits are then selected for the next step. Second, structural assemblies are built using a coarse representation involving only the C-alphas and the centres of mass of the side chains. While fragments are extracted from the best aligned regions of the selected templates, pure ab initio modelling is used to create sections without templates. Fragment assemblies are performed by a modified version of the replica-exchange Monte Carlo simulation technique (REMC) [57] constrained by a knowledge-based force field including PDB-derived and threading constraints, and contact predictions. Generated conformations are then structurally clustered to produce a set of representatives, i.e. cluster centroids. Third, those structures are refined during another simulation stage to produce all atom models. This mixed strategy has proved extremely successful since the “Zhang-Server” [58], which is a combined pipeline of I-TASSER and QUARK (see next section for details), has been ranked as the best server for PSP in several recent CASP experiments [56, 58, 59], when all target categories are considered. However, when only FM targets associated with ab initio approaches are taken into account, Rosetta has often provided more accurate models than I-TASSER [60–63].

Having identified force fields and search strategies as the main limitations to accurate PSP, in 2012 it was proposed to address them by offering a new approach dedicated to ab initio structure modelling, QUARK [24]. It takes advantage of I-TASSER and Rosetta’s strengths: in addition to sequence profile and secondary structure, QUARK also uses predicted solvent accessibility and torsion angles to select, like Rosetta (see next section for details) and unlike I-TASSER, small fragments (size up to 20 residues) using a threading method for each sequence fragment. Then, using a semi-reduced model, i.e. the full backbone atoms and the side-chain centre of mass, and a variety of predicted structural features, an I-TASSER like pipeline is followed: assembly generation using REMC simulations, conformation clustering and production of a few all-atom models. In this phase, not only does QUARK allow more conformational movements than I-TASSER, but also utilises a more advanced force field comprised of 11 terms including hydrogen bonding, solvent accessibility and fragment based distance profile [24]. Although, when QUARK started contributing to CASP9, it was outperformed by Rosetta, positions have been reversed in most subsequent versions [63].

Besides FRAGFOLD, I-TASSER, Quark and Rosetta, there have been a few other predictors, built following the fragment-based paradigm, which present interesting features. ‘Undertaker’ uses fragments of very different lengths excised from three different libraries comprised of (1) generic fragments whose length is 2–4, (2) 9–12 length fragments and (3) fragments of larger size extracted using fold recognition techniques. Sampling is performed using a genetic algorithm [64]. On the other hand, PROFESY adopts a library of 15-residue fragments and the assembly phase is conducted using Conformational Space Annealing [65]. Fragment-HMM is a consensus method; it includes threading, homology modelling, fragment packing, refinement and quality assessment to generate a final candidate model, while position-specific HMM is used to sample the target sequence [44].

### Rosetta

Initially an algorithm for ab initio PSP, Rosetta is currently a macromolecular modelling package comprising programs, scripts and tools for ligand docking [66, 67], protein-protein docking [68], protein design [69, 70], and loop modelling [71]. Rosetta PSP was best for FM targets in CASP12 (2016). It is worth noting that besides the two ‘official’ Rosetta groups, more than 12 participating groups relied on Rosetta in that competition. While Rosetta did not perform as well in CASP13 (5th place), it contributed to the predictions of over 20 groups.

Rosetta relies on fragments of fixed size: 9-mers represent the core of the building process, whereas 3-mers play a refinement role. Those fragments are currently excised from 16,801 high resolution template structures (average size of 257 AAs). The ‘picker’ tool includes the ‘quota protocol’ that is dedicated to pick fragments for ab initio PSP [72]. It guarantees that the selected fragments display the same secondary structure proportions than those predicted by the secondary structure predictors - in our experiments - from three sources, i.e. PsiPred [73], Jufo [74] and SAM [75]. The scoring function, on which the selection of candidate fragments is based, is evaluated at each position in the sequence of interest typically to generate 25 and 200 9-mers and 3-mers respectively. The scoring function is expressed by the following three terms: the PSI-BLAST sequence profile [76], the secondary structure prediction and the Ramachandran map probability value of the segment’s middle residue. The above mentioned secondary structure predictors along with all default “quota protocol” flags described in detail in the literature [72] have been preserved in our standard and customised predictions.

Starting from a fully extended chain, the fragment assembly process takes place via a Monte Carlo search; a sequence window of length 9 is randomly selected and one of the available 25 candidate fragments is randomly selected. Once the torsion angles of that window are replaced by those of the chosen fragment’s, the coarse-grained energy score is calculated; the minimisation process is performed using simulated annealing [14]. Therefore, if either the energy score after an insertion is smaller than that of the previous conformation or, to avoid local minima, the Metropolis criterion is fulfilled for a given ‘temperature’ [77], the substitution is accepted. In simulated annealing, the ‘temperature’ is first set to a high value, then it is decreased, reducing the probability of accepting an energy increasing move. Initially only heavy backbone atoms are considered: after completion of the 9-mer insertion phase that involves 28,000 insertion attempts, a further 8000 insertion attempts using fragments of size 3 are performed creating a coarse-grained conformation. Then, the ‘relax’ phase takes place: all additional atoms are added and fine-tuned using an all-atom or fine-grained energy score. With exception of the number of fragments in the SS-based predictions, no change took place regarding the Rosetta’s ab initio protocol.

### Resampling techniques

Resampling techniques allow narrowing the conformation search space by treating the first round of sampling as ‘draft’ or training predictions so that ‘successful fragments’ can be selected for the following rounds of predictions where experiments focus/exploit deeper specific regions [78–83]. In Rosetta, implementation of Model-Based Search instead of Monte Carlo showed a 14% improvement for the lowest energy models. In that approach, after each iteration, a new search space is defined by selecting, among the regions that can be considered as funnels, the most promising ones according to their shape, size and energy score. In the subsequent iterations, those funnels are explored further by reusing only the fragments from which conformations in those regions were formed [79, 80]. This was further refined by adding additional restrictions by including structural and energy-based information such as the torsion angles, secondary structures and beta pairings that were produced during the first round of predictions [78]. Thus, during the second round, a combination of the frequency of that information along with the low energy scores regions was used to change the criteria that the “Fragment-picker” applied to conduct the fragment selection process. Such approach creates large variations between the fragment sets used during the first and the subsequent rounds. Consequently, not only are the promising funnels already explored in the first turn further exploited, but new regions can be explored by taking advantage of the presence of new fragments. This approached led to improvement averages of 1.7 Å and 0.4 Å in terms of best and best-of-five predictions.

EdaFold, an advanced resampling technique based on Estimated Distribution Algorithm, has been successfully carried out on Rosetta in three releases [82–84]. EdaFold’s main goal is to assign and amend a probability value for each fragment in each subsequent iteration using estimated probability mass functions by further focusing on the fragments found in the basins where a high number of low-energy decoys lies. In its latest release [84], structural dissimilarity was introduced besides energy score as a second criterion for choosing guiding models: fragments that have built those models have their associated probability raised and, consequently, they are picked more often in the next conformation assembly iteration. This addition takes advantage of as many ‘deep funnels’ as possible since from each of those funnels, only one guiding model – the one having the lowest energy score - is chosen. They reported having more than 19% and 8% improvements over standard Rosetta in terms of best and best-of-five predictions respectively.

These studies suggest that the standard number of fragments is much larger than what Rosetta needs in order to reach native-like conformations provided that the search space already explored in the first round contains such a region. All resampling approaches comprise at least a second customised round of sampling, which, therefore, requires more decoys, time and effort; for instance, EdaFold is 2.5 times slower than Rosetta. Moreover, most of the above-mentioned approaches do not search trajectories that go beyond the space reached in the first round: the focus is on promising regions for better exploitation [78].

### Overview

Despite decades of research on sequence-structure mapping [85, 86], no accurate one-to-one mapping function has been discovered even for short sequences. Still, many studies have suggested that its complexity depends on the associated secondary structure [87, 88]. Indeed, while alpha helix fragments used for fragment-based PSP have revealed a relatively limited diversity [89], research in loop modelling has highlighted not only the diversity of those structures, but also the influence of many factors such as length, anchor region and even external interactions with the environment [87, 88, 90–96]. Recently, a new fragment builder called “Flib” has been proposed [89], where fragments are created within the 6 to 20 residue range. Their selection relies in particular on their dominant predicted secondary structures, i.e. α-helical, β-strand, loop and neutral. While Flib’s α-helical and β-strand fragments are state-of-the-art, where the α-helical ones are the most accurate, loop dominated fragments remain a real challenge. Taking also advantage of secondary structure information by relying on the reliable sequence-based structural class prediction, a customised fragments’ library was created by restricting the set of structures where those fragments are extracted from [32]. Such library was able to improve quality of both decoys and first models. Based on a similar concept, a methodology was introduced later on to decrease the number of 3-mers – that are responsible for both minor conformational changes and corrections for specific proteins that belong to certain structural classes [38]. Likewise, improvement was shown, however, in terms of first models only: indeed Rosetta wasn’t able to discover new search area but rather focused on specific regions where diversity of 3-mers were likely to damage alpha and alpha-beta proteins’ final conformation predictions.

In this study, we investigate further the optimisation of fragment usage. However, instead of changing the library of structures where fragments are selected from or the criteria of selection, here it is proposed to set the number of available candidate fragments for each position according to the secondary structure annotations that the fragment – either 9-mer or 3-mer – is likely to adopt. Such methodology takes advantage of strong empirical previous studies (presented in the previous section); for instance, since it was shown that a set of alpha helix fragments is unlikely to comprise outliers, a subset of them or even a single randomly selected one should be “good enough” [89]. Thus, it is proposed to optimise the space search by dedicating more time on exploiting relatively small areas in the search space. A strength of such approach is that it does not require any resampling overhead as described above in methodologies relying on excluding unnecessary fragments.

For example, if an amino acid belongs to an alpha helix, a single 9-mer is used instead of a set of 25, selecting the fragment that has the highest score according to fragment picker; a similar policy is applied to only consider the top 5 fragments out of the 200 in the 3-mer file. Similarly, in the case of a beta strand, the number of 9-mers is kept to 25, whereas the number of 3-mers is reduced to the top 25. On the other hand, whenever there is a coil, the standard number of 9-mers, i.e. 25, and 3-mers, i.e. 200, are used to maintain structural diversity in amino acid sequences predicted to adopt a coil configuration.

Herein, we conduct a thorough study and evaluation of this new methodology on a set of 24 targets belonging to different structural classes and whose length reaches up to 150 amino acids. We have adopted such protein size threshold due the performance abilities of Rosetta [82, 97, 98]. First, we present the dataset used besides and the size of both new fragment files. Second, we show detailed results of the blind assessment – the one adopted in CASP – comparing Rosetta’s standard and new approaches using a large number of decoys. Third, we discuss the results of the “best decoy” (regardless of the energy score). Finally, we describe the proposed fragment-based protein structure prediction methodology.

## Results

### Experiments and dataset

In order to validate the proposed methodology, Secondary Structure-based Rosetta (SS-Rosetta), where the number of fragments is customised for each amino acid, for each target, its performance is compared to a standard version of Rosetta. In this manuscript, standard Rosetta is defined by the usage of the latest Rosetta ab initio protocol (version 3) that can be downloaded from https://www.rosettacommons.org/, using default parameters, tools and databases. More specifically, the Rosetta ab initio protocol used in this study is version 3.4. Fragments were generated by the fragment picker that Rosetta has been using since 2011 with the quota protocol that was designed for ab initio PSP [72]. The database of protein structures from which fragments are extracted – nicknamed vall – is the latest version (July19.2011) that contains 16,801 template structures. None of the parameter values proposed in the quota protocol [72] has been altered (see the exhaustive list and command line in supplementary material).

The only difference between ‘standard’ Rosetta predictions and SS-Rosetta is that instead of relying on 25 9-mers and 200 3-mers, which are the default numbers of fragments that have adopted by Rosetta for more than 15 years [72, 99, 100], SS-Rosetta customises them according to the target. Whilst the whole fragment selection process has not been altered, the number of fragments at specific positions is changed. It is worth noting that SS-Rosetta produces decoys at the same speed as standard Rosetta.

Although, in practice, a secondary structure predictor should be used to provide the required secondary structure information, here the secondary structure annotations associated to each target are exploited so that the evaluation of the proposed approach is independent from the choice of a predictor and its accuracy. However, since such predictors already provide predictions with an accuracy above 80% [101, 102] and it is constantly improving, usage of actual secondary structure information should not affect this study’s conclusions.

For this experiment, two sets of decoys are used: 20,000 and 2000. Although, it is recommended to produce as many decoys as possible, 20,000 is considered an appropriate number to fairly assess Rosetta’s performance [82, 97, 103, 104]. Whilst such a large number of decoys usually requires usage of parallel computing, the production of 2000, on the other hand, could be processed by a standard PC [104]. The rationale behind using both a large and a relatively small number of decoys is to investigate how that number impacts the exploration/exploitation compromise and to evaluate the performance of our proposed methodology when implemented on either a high-performance computing system or a typical PC.

As shown in studies conducted by the EdaFold’s team [82–84], strategies for enhancing and/or optimising fragments in Rosetta can be rigorously evaluated by using a 20 protein dataset that shows diversity in terms of percentage and length of helices and sheets. Since the proposed approach relies on the secondary structure of a protein target, it is important for the evaluation of our approach that the test dataset presents proteins with a variety of secondary structures. As the original 20-target set did not offer enough diversity, e.g. the all-alpha class was only represented by 3 proteins, and their average sizes were quite far from the 150 amino acid threshold (the largest target had a size of 128, while the average size was 86), a small number of additional targets (5) were added to the original set to make it more balanced and including larger targets (the largest target has now a size of 149, while the average size was raised to 89). To make sure that those targets were independently annotated as FM hard targets, we took advantage of past CASP experiments, which has a rigorous process to classify targets in the FM hard class. Therefore, three criteria were used to select those additional targets: their annotation as FM, their secondary structure and their length, in line with the 20 FM protein dataset constraints, i.e. shorter than 150 amino acids. As that set was designed to offer targets of average complexity, we decided to add of a few hard targets* to allow better evaluation of SS-Rosetta. Note that as all homologs (including potential remote homologs) are removed from the fragment libraries, the edition of CASP from which targets are coming does not affect their complexity as seen by either Rosetta or our enhanced version. This was the reason why targets were originally only defined by their PDB id. However, for completeness, CASP Identifier, CASP round and their individual performance in our experiments have now been included for those targets in the supplementary material (Table ST1). Since one of the targets (1BQ9) is classified structurally as a coil-based small protein, it is out of the scope of proteins whose predictions can be enhanced by our approach. In practice, our approach would simply apply the standard Rosetta process on it. Therefore, its inclusion in the set would not provide any useful information. Eventually, an updated dataset of 24 proteins with lengths in the 56 to 149 range was created: 5 FM hard targets* were added, 3 of them in the all-alpha class as it was underrepresented (only 3) in the original 20-target set. The whole dataset is shown Table 1, where the last 2 columns report the size of the new fragment files (corresponding to the number of fragments used for each target) as a percentage of their initial sizes.

**Table 1:** The 24 protein targets included in this study

#	PDB ID	Length	% of helices	% of strands	% of coils	Relative size of the new 9-mer file	Relative size of the new 3-mer file
1	2CI2_I	65	16	21	63	85.8%	51.7%
2	1CTF	68	51	24	25	85.2%	49.7%
3	1DI2	69	46	33	21	89.5%	47.8%
4	1SCJ_B	71	23	39	38	86.5%	49.5%
5	1HZ5	72	30	38	32	79.2%	37.0%
6	1CC8	72	28	35	37	88.2%	55.3%
7	3NZL	73	59	0	41	79.5%	41.4%
8	1DTJ	74	38	26	36	88.4%	51.8%
9	1IG5	75	61	5	34	83.5%	47.6%
10	1OGW	76	25	34	41	81.4%	47.9%
11	1DCJ	81	28	24	48	77.8%	40.5%
12	1TIG	88	32	32	36	50.0%	29.7%
13	1A19	89	43	17	40	92.9%	61.5%
14	1BM8	99	37	27	36	93.9%	58.4%
15	4UBP	100	54	17	29	77.2%	46.5%
16	1IIB	103	55	19	26	94.9%	73.4%
17	1M6T	106	77	0	23	84.8%	50.1%
18	1ACF	125	34	32	34	77.8%	45.1%
19	3CHY	128	45	17	38	73.9%	45.3%
20	2KDL*	56	62	0	38	87.7%	50.2%
21	2LR8*	70	57	0	43	74.0%	44.0%
22	4HLB*	95	28	24	48	78.2%	49.6%
23	2K4V*	125	28	32	40	72.1%	48.4%
24	2KY4*	149	59	1	40	87.9%	54.1%
**Average**		**88.7**	**42.3**	**20.7**	**37.0**	**82.1%**	**49.0%**

* Not only did those targets prove particularly difficult to predict during CASP, but the Global Distance Test (GDT) of the best decoys (out of 20,000) in standard predictions is significantly lower (~ 50) than the GDT of the other targets of similar length and confirmed by Table ST1, the 5 added CASP targets create a more challenging set: the average GDT of their best decoy out of 20,000 (regardless of the energy score) is below 50 (i.e. 47), which is the threshold usually used to qualify an alignment as ‘good’ [34]. Moreover, as the table shows their associated first, best and average of the best 5 models are particularly poor for all targets except 2LR8

Performance evaluation is conducted in two parts: i) as CASP evaluates competitors’ models, a blind assessment compares the first models, i.e. the conformations that correspond to the lowest energy scores of each set of decoys, and ii) the best decoy’s quality regardless of the corresponding energy scores.

### Blind assessment

**Fig. 1:**
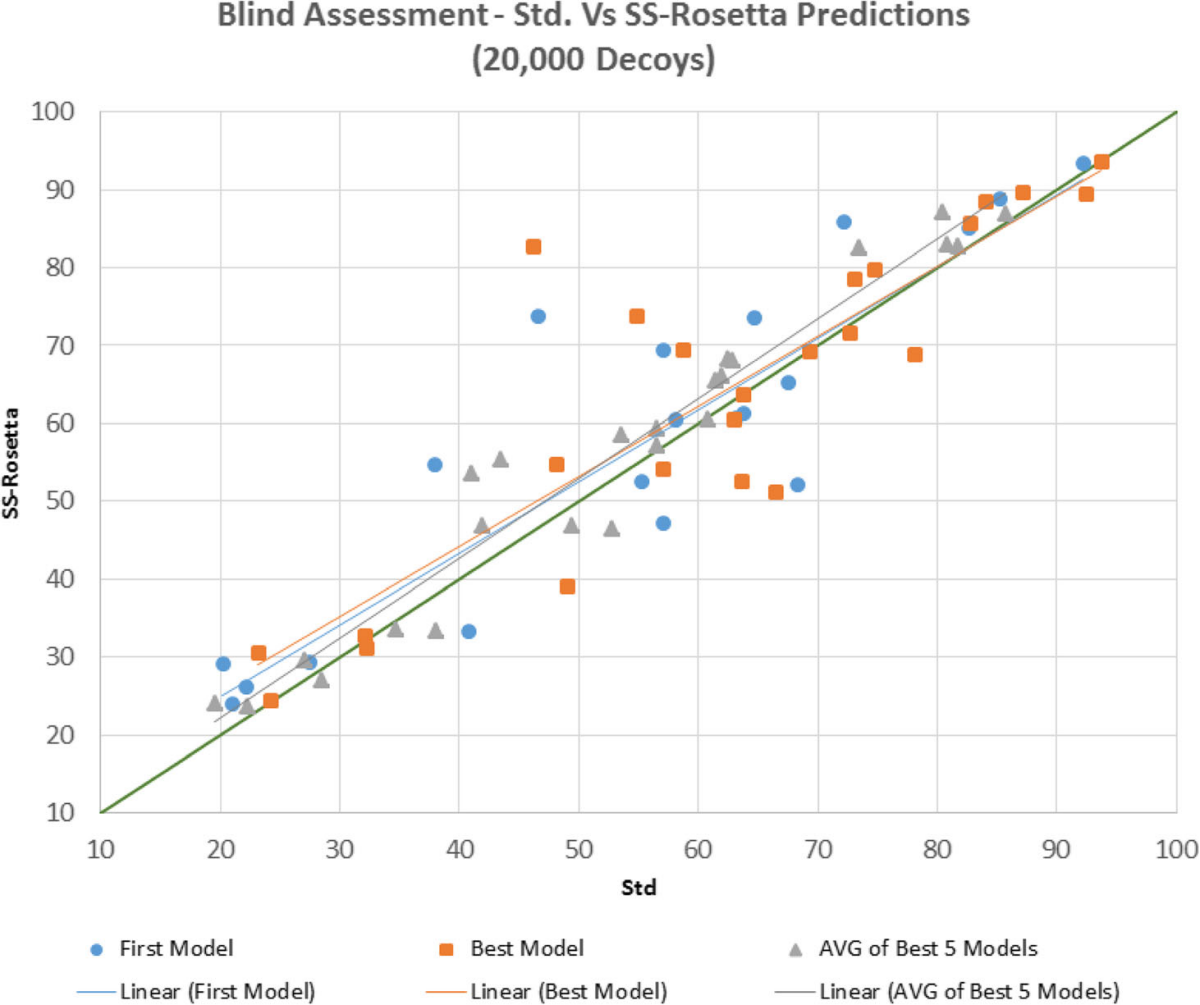
GDT’s of First, Best and Top 5 models for standard (denoted as ‘Std’) and SS-Rosetta predictions for 20,000 decoys. Linear regression lines are shown for the three scores

**Fig. 2:**
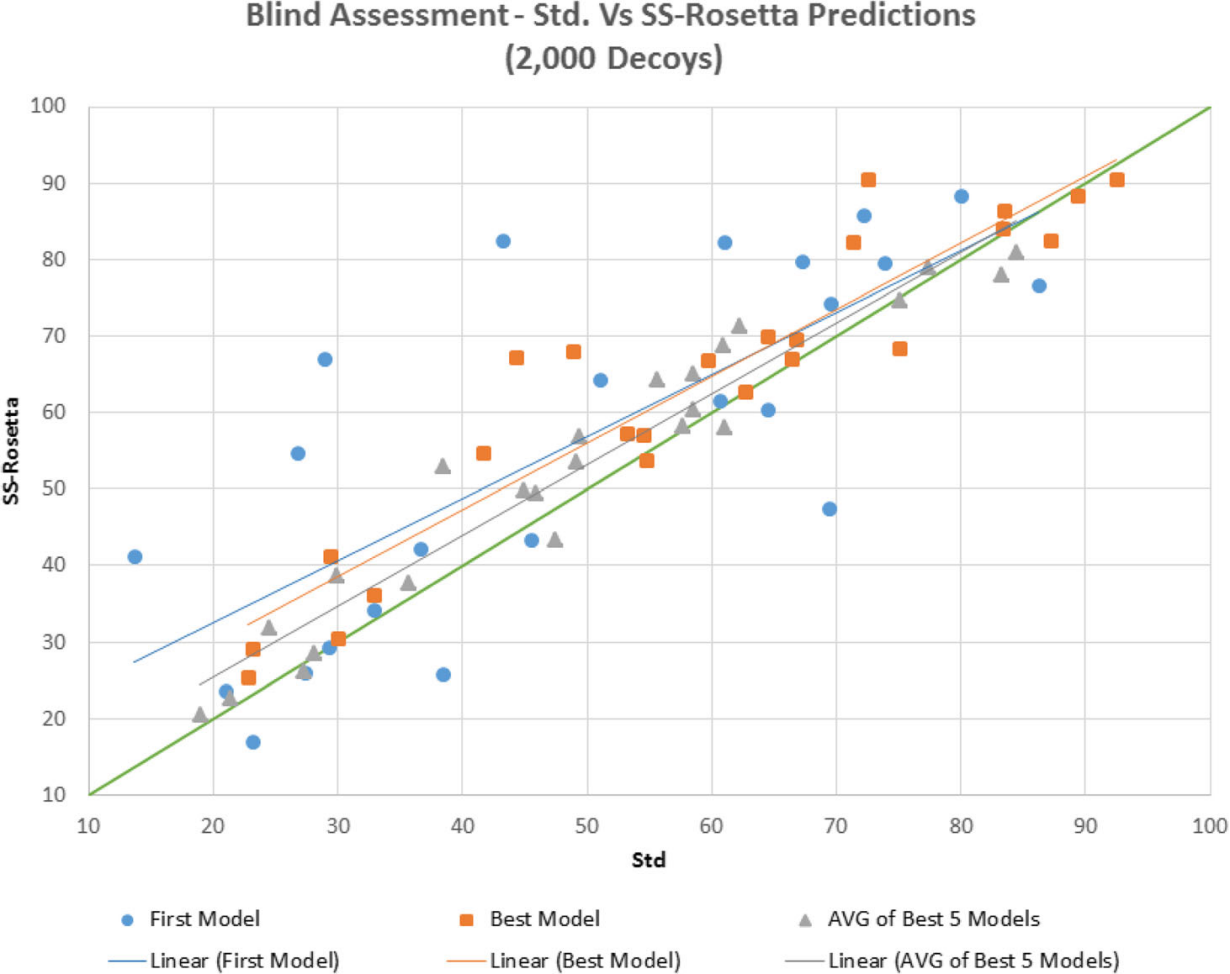
GDT’s of First, Best and Top 5 models for standard (denoted as ‘Std’) and SS-Rosetta predictions for 2000 decoys. Linear regression lines are shown for the three scores

**Fig. 3:**
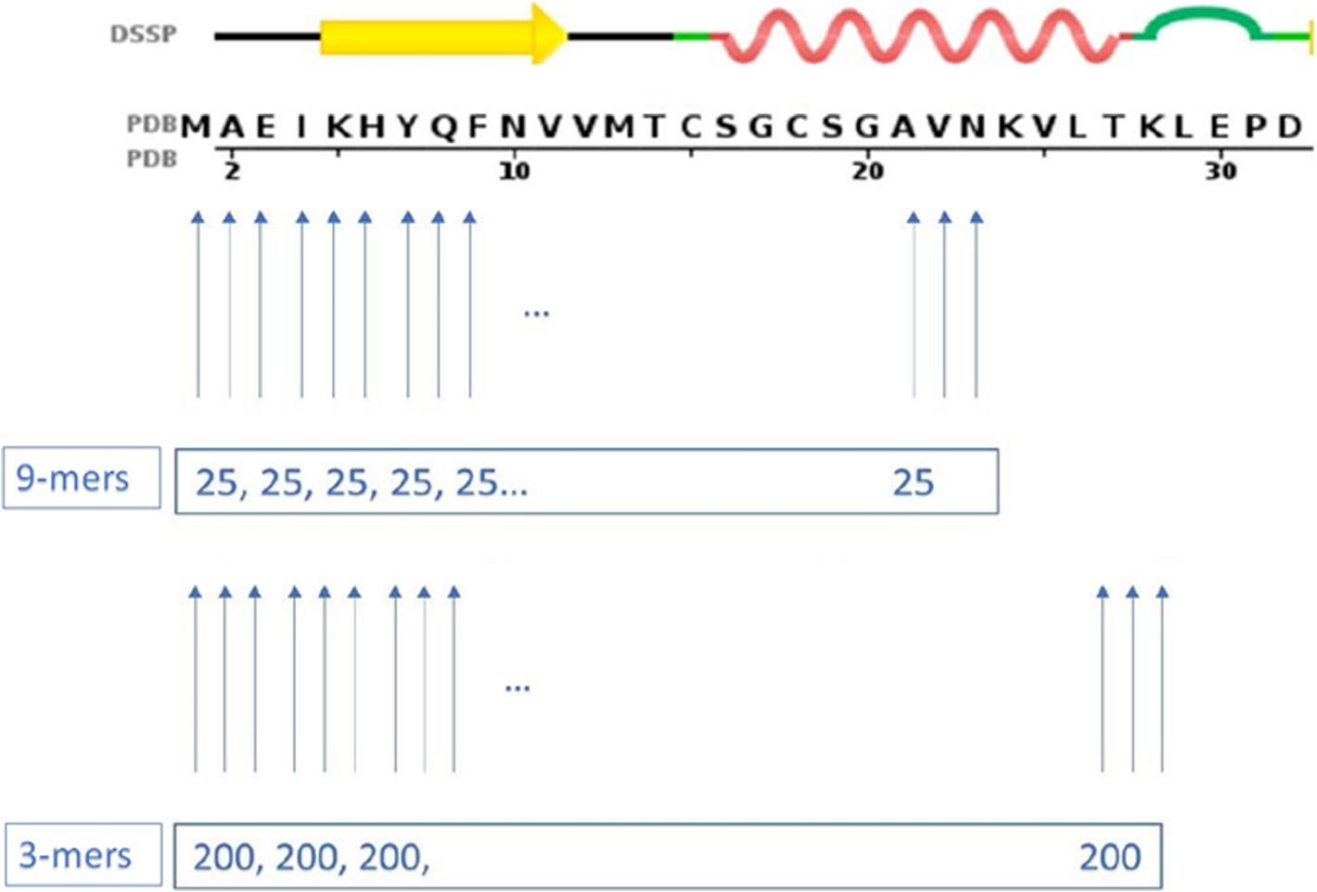
A pictorial representation of the number of fragments contained in Rosetta standard’s 9-mer and 3-mers files (the upper part of the figure was taken from the PDB, sequence tab of the Atx1 Metallochaperone Protein – PDBID: 1CC8). In the case of 9-mers, the blue arrows point to the positions where there is a set of 25 candidate fragments of length 9. In the example above, assuming that the protein is of length 32, the 9-mer fragment library ends at position 23. In the case of 3-mers, there are 200 candidate fragments per position. Here the 3-mer fragment library ends at position 30

**Fig. 4:**
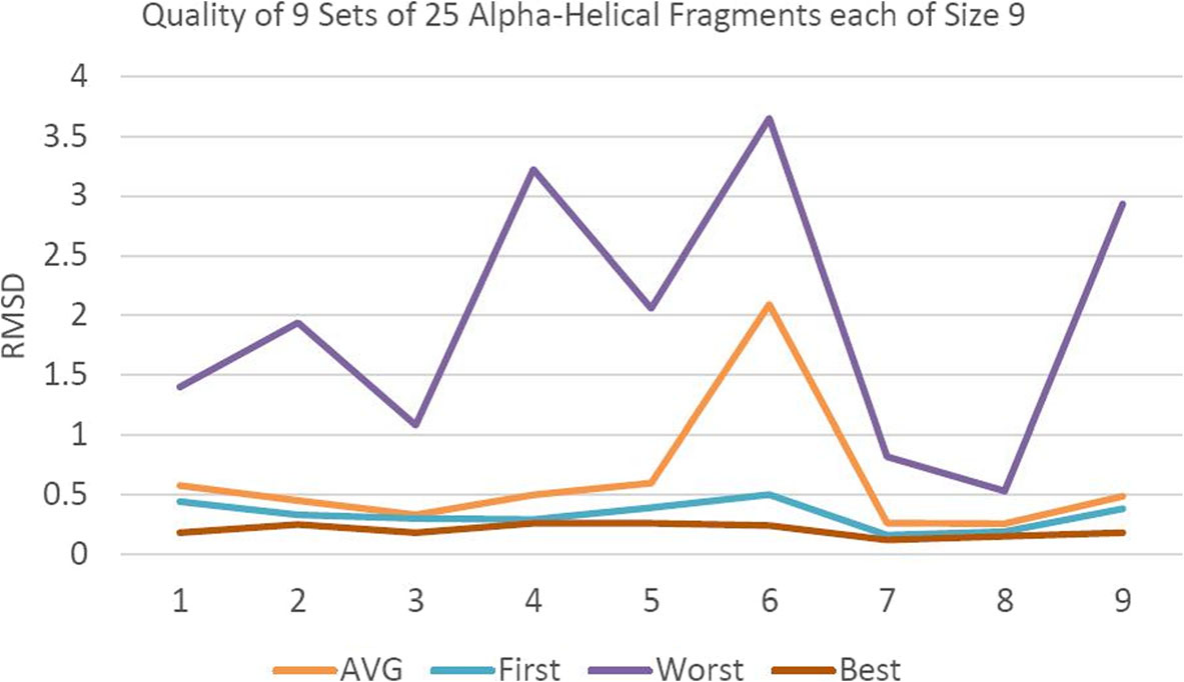
RMSD of 225 9-mers distributed amongst 9 positions as averages, lowest, highest and first ones

Blind assessment mimics CASP’s assessment as groups can submit up to 5 models and should designate one of them as the first model. The two main ranking scores adopted are the GDT of the first model as well as the Best model – the best of the 5 submitted structures. Furthermore, we introduce here a third criterion too - the average of the 5 models - to shed light on the quality of the models that lie in the lowest 5 positions in the search space. Figures 3 and 4 compare the First, Best and Top 5 average of the models produced by standard and SS- Rosetta for both 20,000 and 2000 decoys respectively. When 20,000 decoys are generated (Fig. 1), out of the 24 targets, SS- Rosetta produced 15 better First models (+ 6.3% average GDT), 12 better and 3 equal Best models (+ 5.0% average GDT), and 18 better Top 5 models (+ 6.1% average GDT). SS- Rosetta’s performance is even more remarkable in the 2000-decoy experiment (Fig. 2), where GDT gains are further amplified reaching 24.2% for the First models. All quantitative values are presented in Table 2.

**Table 2:** Blind assessment showing improvements of SS-Rosetta’s performance against standard predictions’ in terms of number of better structures (out of 24) and GDT

Std vs SS- Rosetta	*First model*	*Best model*	Average of the *Best 5 models*
20,000 Decoys	15/24 (63%) + 6.3%	12/24 (50%) + 5.0%	18/24 (75%) + 3.1%
2000 Decoys	16/24 (67%) + 24.2%	18/24 (75%) + 11.5%	18/24 (75%) + 8.3%

As mentioned previously and illustrated by the results displayed in Table 3, the higher the number of decoys, the higher the probability to hit a better conformation. As the comparison with Rosetta’s standard predictions shows (see Table 2), our proposed pipeline dramatically improves predictions for the 2000-decoy experiments (+ 24% for *First model*), whilst still enhancing predictions (+ 6%) in the 20,000-decoy experiments. When the number of decoys is small, further exploration is more valuable than additional exploitation; this is due to the nature of random trajectories – an outcome of the random choice of a large number of available fragments at random positions - that are likely to be quite different. When the number of available fragments sharply decreases, search trajectories tend to focus on limited regions, further exploiting funnels which allows discovering a larger number of local minima.

**Table 3:** Performance comparison between generating 20,000 and 2000 decoys in both standard and SS-based predictions

	*First model*	*Best model*	Average of the *Best 5 models*
Std. Predictions 2000 vs 20,000 Decoys	17/24 (71%) + 26.3%	19/24 (79%) + 7.6%	19/24 (79%) + 8.2%
SS-based Predictions 2000 vs 20,000 Decoys	18/24 (75%) + 30.2%	19/24 (79%) + 11.7%	19/24 (79%) + 14.7%

Thus, we formulate the hypothesis that our approach discards unhelpful fragments and the known inaccuracy of energy functions suggest that the production of those larger numbers of local minima could be the source of those improvement in terms of “First Model” for the 2000-decoy predictions. To support this hypothesis, another experiment was conducted to compare standard predictions, for both 2000 and 20,000, according to the quality of the best decoy regardless of its energy score. It shows that for all targets the 20,000-decoy experiments reached a more accurate decoy. Whilst for the Best model improvement reached over 26% as shown in Table 3, the quality of the best decoy only increased by 6%.

While production of a high number of decoys is suitable, the associated computation cost, typically a few minutes are required to generate a single decoy on a standard PC, is often impractical: conducting a standard Rosetta model prediction with its recommended 20,000 decoys may require weeks of computations on a PC. As a consequence, pragmatically, a reduced number of decoys is often used despite the associated impact on the quality of the generated models. As Table 4 shows, SS-based delivers predictions with 2000 decoys that match the accuracy of those produced by standard Rosetta with 20,000 decoys. The associated reduction of processing time makes production of quality models using Rosetta much more accessible.

**Table 4:** Blind assessment results of standard predictions’ 20,000 decoys against SS-based predictions’ 2000 decoys. Although our approach shows slightly better outcomes, there is no significant difference between the two sets of results

	*First model*	*Best model*	Average of the *Best 5 models*
20,000 Decoys (Std. Predictions) vs 2000 Decoys (SS-based. Predictions)	12/24 (50%) + 0.4%	13/24 (54%) + 4.6%	10/24 (42%) + 0.3%

## Discussion

Presented results support the hypothesis that the redundancy of the fragments of Rosetta’s standard predictions makes the trajectory paths explore a large space on the energy landscape preventing the exploitation of promising funnels. This is addressed by making the process of searching the conformation with the lowest energy score less random. The focus of the new approach is on investigating the most challenging structural areas like coils’, limiting, in particular, the consideration of helical regions that have low structural diversity. Improvements of *first and best models*, 24% and 11.5%, respectively, in terms of GDT, validate such strategy. In addition, although the standard approach explores a larger search space, it is not able regardless of the energy score, i.e. in terms of *best-decoy*, to locate better conformations than those produced by the proposed approach.

As all fragment-based PSP tools suffer from excessive processing times, a particularly beneficial outcome delivered by this research is their dramatic reduction, i.e. by a factor 10. Indeed, we have shown that our approach, using only 2000 decoys can produce results as accurate as those of the standard approach when using 20,000 decoys. This would not only save processing time, but also allow to democratise fragment-based PSP since predictions could be performed on a standard PC. In principle, the presented approach is applicable to any fragment-assembly PSP tool: whatever the fragment size, their cardinalities could be tailored based on the dominant types of secondary structure.

## Conclusions

We have presented in this paper a comprehensive investigation of the fragment insertion process, which is at the core of fragment-based protein structure prediction. Our study shows that this process should not be treated evenly on all regions of the conformation being built, since the strength of the subsequence-substructure relationship varies according to the type of secondary structure. Indeed, a limited number of selected fragments appears to be sufficient to build the easier regions. This paper’s findings have introduced a new concept during fragment-assembly: the number of available fragments should be set according to the dominant secondary structure of the parts where fragments are to be inserted.

Using Rosetta, 24 targets of different length and structural class – mainly alpha, mainly beta and alpha beta, were extensively examined through two experiments using either 20,000 or 2000 decoys. Whereas the first one revealed improvements of 6.3% in terms of *first model*, it was quadrupled in the second one. Furthermore, it was shown that increasing the number of decoys – the rule of thumb in fragment assembly predictions to further explore/exploit the search space - would have more effect using our proposed technique rather than the standard one.

Owing to the high accuracy of secondary structure predictions we believe that redundancy of fragments should be relative to the dominant type of the secondary structure of the substructure being amended. Dedicating more ‘effort’ on coils and beta sheets leads to a substantial enhancement that would be worth taking into account in all fragment assembly approaches.

## Methods

### Proposed methodology

To our knowledge, none of the past studies aiming at enhancing Rosetta has considered using a variable number of fragments during its 3-mer and 9-mer fragment insertion processes. Owing to the strong sequence-structure relationship for alpha helices, the weaker one for beta strands and the loose one for coils, a novel approach is proposed where the number of available fragments per position varies according to its associated predicted secondary structure.

To implement this, it is important to identify an appropriate number of fragments for 9-mers and 3-mers for each of the three different secondary structures. This was performed empirically using a representative protein sequence, i.e. a target whose secondary structures are relatively evenly distributed and which can be classified as being of ‘average’ complexity. As 1CC8 contains 28% alpha helices, 35% beta sheets and 37% coils, and the best predicted model by standard Rosetta had a GDT of 55, which suggests an ‘average’ complexity, that target was selected.

Standard Rosetta associates to each amino acid of the structure to be predicted fragment files with fragments of length 9 and 3. In the 9-mer insertion phase, 25 fragments are selected starting at the first amino acid till the position: ‘total_size - 9’. Similarly, in the 3-mer insertion phase 200 fragments are selected starting at the first amino acid till the position: ‘total_size - 3’. Fig. 3 displays the number of fragments contained by the fragment files associated to the 32 first residues of the sequence of 1CC8.

**Fig. 5:**
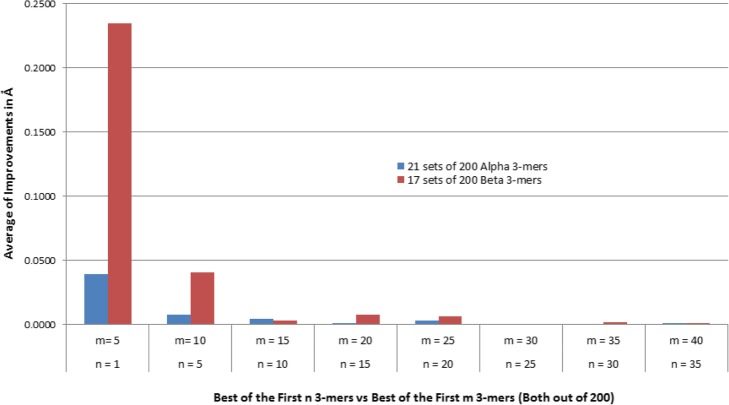
Average RMSD gain when using the best fragment from a set of size m instead of a smaller set of size n. New fragments are appended to the set based on the score generated by the “Fragment-picker”

That sequence has 9 positions where the 9-mer belongs to a pure alpha helical structure. For each of those 9 positions, the Root Mean Square Distances (RMSD) of the average, lowest (best), highest (worst) and first of the 25 fragments are plotted in Fig. 4. It shows that, for the 9 positions, the first fragment in the set of 25 is very close to be the best, better than the average fragment and much better than the worst one. As a consequence, replacing the 25 fragments by the first one has the potential to lead to better structure prediction than by relying on a random process. Similarly, our study evaluated the quality of the 21 and 17 positions where 3-mers belong to a pure alpha helical and a pure beta strand respectively. In order to set an adequate minimum number of fragments at those positions, we have conducted a thorough study on the average RMSD gain when using the best fragment out of a set of 5, 10, 15, 20, 25, 30, 35 and 40 (Fig. 5).

Figure 5 shows that usage of only the first fragment would not be a sensible choice since important improvements (11.1% and 27.3% for alpha helix and beta sheet 3-mers respectively) occur when the number of fragments is increased to 5. Beyond the first 25 fragments, improvements become negligible (< 0.2%) for both types of fragments. Moreover, in the case of alpha helical 3-mers, since usage of more than 5 fragments brings an improvement of less than 1.5%, the number of fragments could be set at 5 for that category. On the other hand, the quality of beta 3-mers still improves significantly (+ 4%) with 10 fragments and keeps increasing (an additional 1.5%) until the top 25. Thus, the number of fragments for amino acids predicted to belong to beta sheets could be set at 25.

**Fig. 6:**
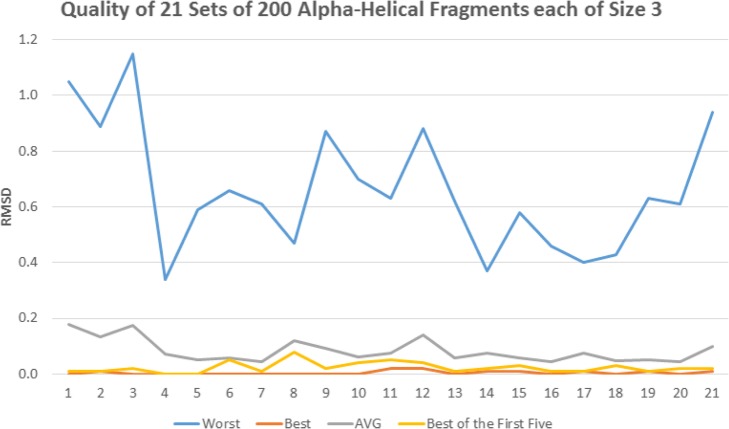
Quality of 4200 3-mers at 21 positions as RMSD of their averages, lowest, highest and first ones

**Fig. 7:**
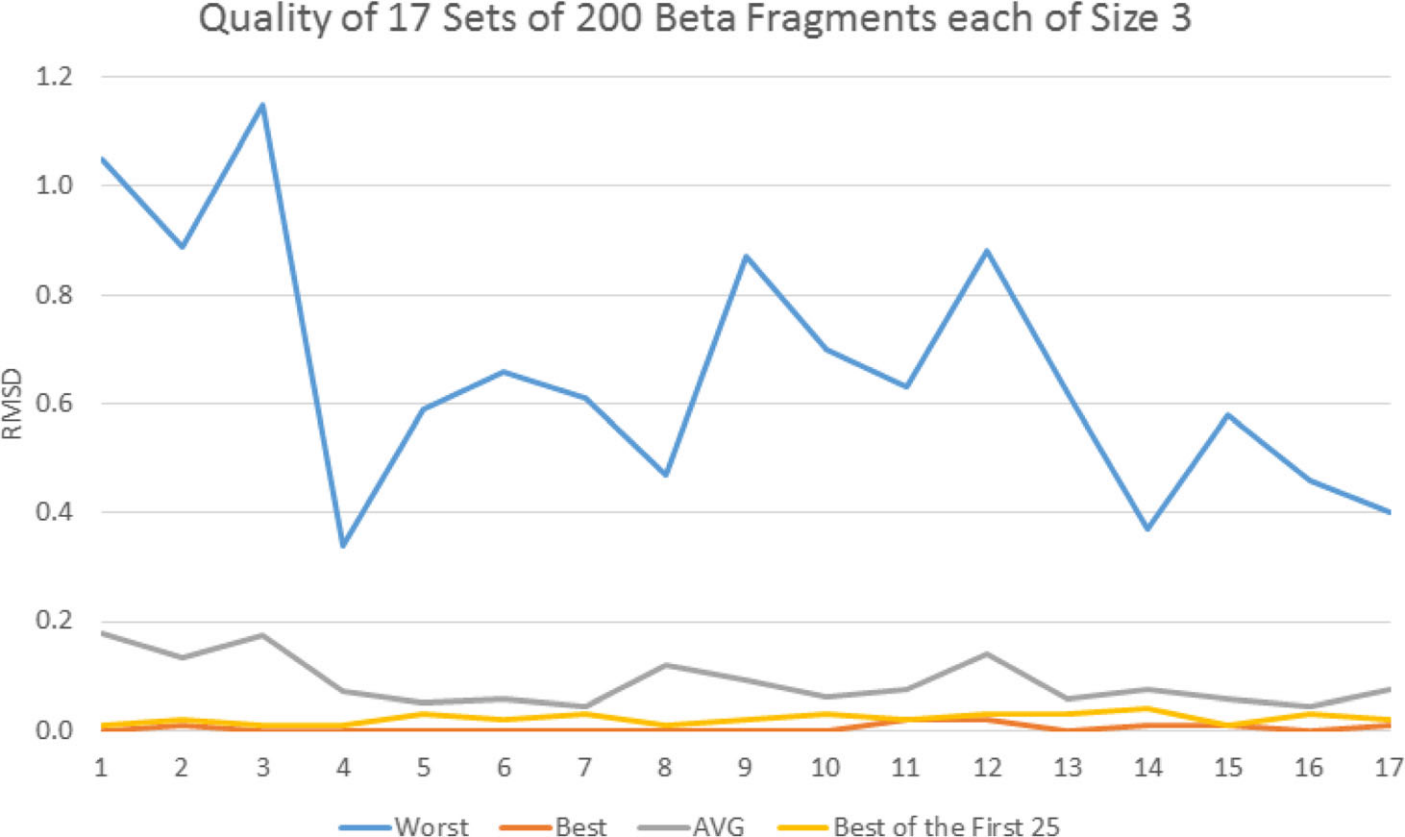
Quality of 3400 3-mers at 17 positions as RMSD of their averages, lowest, highest and first ones

Further investigation was conducted: out of the 200 3-mers, the lowest (best), highest (worst), average and the lowest RMSD of the first 5, 25 respectively, fragments are plotted in Fig. 6, Fig. 7 resp., where a 3-mer is predicted to be an alpha helix, beta strand resp. The best fragment (out of 5 or 25) is very close to the whole set’s best one (out of 200) and much better than the average. Accordingly, the numbers of fragments were set to 5 and 25 for 3-mers that are predicted to be alpha helices and beta strands respectively.

**Fig. 8:**
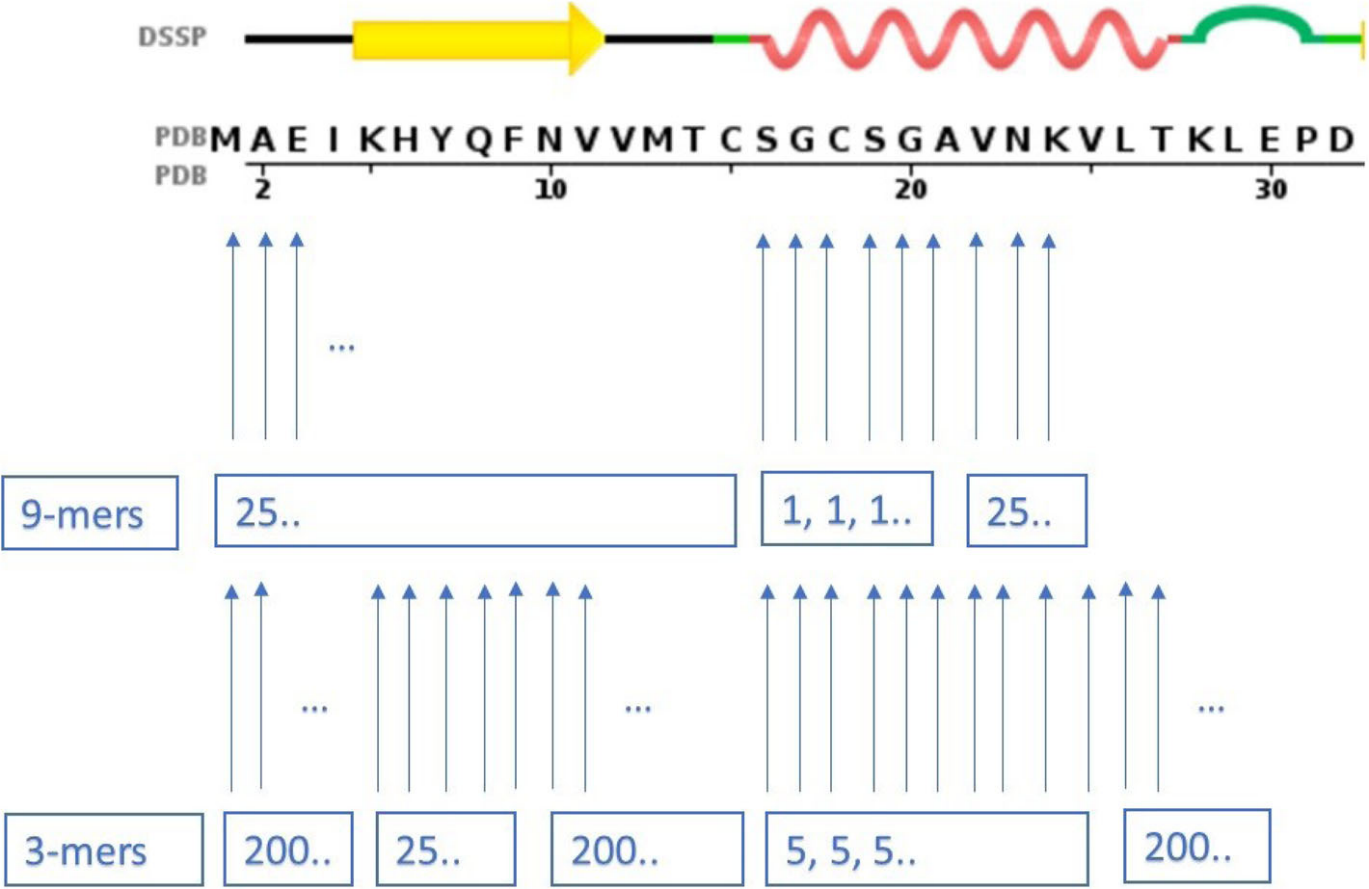
A pictorial representation of the number of fragments contained in SS- Rosetta’s 9-mer and 3-mer files. For the 9-mers, since at positions 16, 17, 18, 19, 20 and 21 a helix of size 9 is predicted, only one fragment is used. The standard number of fragments, i.e. 25, is kept for all remaining locations. For the 3-mers, since at positions 4 to 9 a strand of size 3 is predicted, only 25 fragments are used, and at positions 16 to 25 a helix of size 3 is predicted, only 5 fragments are used. The standard number of fragments, i.e. 200, is kept for all remaining locations

Consequently, application of the proposed methodology to both fragment files should lead to a dramatic reduction of the number of candidates whenever the fragment is predicted to be either a helix or a beta strand. A pictorial description is shown in Fig. 8. The process is the following: in the 9-mer file, whenever 9 consecutive amino acids are predicted to belong to a helix, only a single structural fragment is made available for insertion, otherwise, as in standard Rosetta, 25 different ones can be selected. Similarly, when building the 3-mers file, whenever 3 consecutive amino acids are predicted to belong to a helix or a beta strand, only five, respectively 25, structural fragments can be inserted. In all remaining positions, the standard number of available fragments, i.e. 200, is used.

### Evaluation framework

In order to evaluate the proposed methodology, predictions have to be performed using protein sequences of known structure. Since this simulates ab initio protein structure prediction, it is important to make sure that information about any potential homologous structures are excluded from the process of fragment extraction. As a consequence, Rosetta’s ‘default’ ab initio predictions’ policy was followed: during the fragment building process where all proteins that are classified as homologous are excluded from the database of protein templates known as “vall”. Such a classification is mentioned in Baker lab’s de novo key experiments [103] and entails removing all proteins that appear in the result of running PSI BLAST of the protein target against the database of templates using an E-value threshold of 0.05.

### Evaluation metrics

The main metric used to assess the proposed structure prediction pipeline is the global distance test-total score (GDT_TS) – referred simply to GDT. It was introduced as a part of the LGA (Local Global Alignment) method and since then it has been widely accepted in the community mainly due the fact it is less sensitive to outliers than RMSD [105]. GDT is the formal criterion in CASP, and it is defined as the average of the percentage of residues that are less than 1, 2, 4, and 8 Å. For the superimposition of fragments, the standard metric, i.e. RMSD, was used instead of GDT since, for such short sub-structures, not only do outliers no longer exist, but also GDT scores are not able to discriminate between superimposition variations that are lower than 1 Å, which is particularly an issue when dealing with short peptides. All measurements were generated using MaxCluster, a tool for protein structure comparison and clustering [106].

## Supplementary information

**Additional file 1: Table ST1**: Additional information of the 5 CASP targets added to the original dataset. Detailed information regarding parameters, flags and command line used to run ‘standard’ Rosetta.

## Data Availability

Rosetta package can be downloaded for educational purposes for free at https://www.rosettacommons.org/ All files and steps needed to carry on both standard and SS-based Rosetta predictions are available online at https://github.com/JadAbbass/SS-Rosetta The datasets used and/or analysed during the current study are available from the corresponding author on reasonable request.

## Abbreviations

PDB: Protein Data Bank
CASP: Critical Assessment of protein Structure Prediction
TBM: Template-Based Modelling
FM: Free modelling
SS-Rosetta: Secondary Structure-based Rosetta
GDT: Global Distance Test
RMSD: Root Mean Square Deviation
