# Supplementary material for: Enhancing fragment-based protein structure prediction by customising fragment cardinality according to local secondary structure
Source: BMC Bioinformatics. 2020 May 1;21:170. doi: 10.1186/s12859-020-3491-0 (PMC7195757; doi:10.1186/s12859-020-3491-0)
Supplement: Supplementary file 1 — Additional file 1: Table ST1: Additional information of the 5 CASP targets added to the original dataset. Detailed information regarding parameters, flags and command line used to run ‘standard’ Rosetta. [file 12859_2020_3491_MOESM1_ESM.docx]

| PDB ID/CASP Identifier/CASP round | Length | First Model (Standard Rosetta Vs SS-Rosetta) | Best Model (Standard Rosetta Vs SS-Rosetta) | Average of the Best  5 models | Best Decoy out of 20,000 (regardless of the energy score) |
| --- | --- | --- | --- | --- | --- |
| 2K4V/T0482/CASP8 | 125 | 27.40 Vs 29.40 | 32.20 Vs 31.20 | 28.48 Vs 27.00 | 37.60 |
| 2KDL/T0498/CASP8 | 56 | 20.98 Vs 24.11 | 24.11 Vs 24.55 | 22.23 Vs 23.66 | 38.39 |
| 2KY4/T0553/CASP9 | 149 | 20.13 Vs 29.20 | 23.15 Vs 30.54 | 19.53 Vs 24.13 | 41.11 |
| 2LR8/T0665/CASP10 | 70 | 68.21 Vs 52.14 | 69.29 Vs 69.29 | 61.36 Vs 65.50 | 70.00 |
| 4HLB/R0020/CASP ROLL | 95 | 22.11 Vs 26.32 | 32.11 Vs 32.90 | 27.05 Vs 29.63 | 48.16 |

Table ST1: **Additional information of the 5 CASP targets added to the original dataset**

**Detailed information regarding parameters, flags and command line used to run ‘standard’ Rosetta**

(without any loss of generality information is presented for target ‘2CI2’)

“Quota-protocol.flags” file:

# Input databases

-in::path::database rosetta3.4/rosetta_database/

-in::file::vall rosetta3.4/rosetta_tools/fragment_tools/vall.jul19.2011.gz

# Query-related input files

-in::file::fasta 2CI2.fasta

-in::file::checkpoint 2CI2.checkpoint

-in::file::s 2CI2.pdb

-frags::ss_pred 2CI2.psipred_ss2 psipred 2CI2.SAM_ss2 sam 2CI2.jufo_ss jufo

# the name root for the output fragment files

-out::file::frag_prefix Output/frags

# Show score components for each selected fragment

-frags::describe_fragments Output/frags.fsc

# Weights file

-frags::scoring::config quota-protocol.wghts

# we need nine-mers and three-mers

-frags::frag_sizes 9 3

# Select 200 fragments from 1000 candidates. We need more candidates than fragments

# to fill quota pools.

-frags::n_candidates 1000

-frags::n_frags 200

# Quota.def file defines the shares between different quota pools. The total should be 1.0

-frags::picking::quota_config_file quota.def

# Get rid of homologues fragments

-frags::denied_pdb 2CI2.homolog_nr

“Quota-protocol.wghts” file:

# score name priority wght min_allowed extras

SecondarySimilarity 350 1.0 - psipred

SecondarySimilarity 300 1.0 - sam

SecondarySimilarity 250 1.0 - jufo

RamaScore 150 2.0 - psipred

RamaScore 150 2.0 - sam

RamaScore 150 2.0 - jufo

ProfileScoreL1 200 1.0 -

PhiPsiSquareWell 100 0.0 -

FragmentCrmsd 30 0.0 -

“Quota.def” file:

#pool_id pool_name fraction

1 psipred 0.6

2 jufo 0.2

3 sam 0.2

Command line:

mpirun -np 16 /shares/hpc/OS/sles/11_2/rosetta/3.4.0/SRC/rosetta_source/bin/AbinitioRelax.mpi.linuxgccrelease -in:file:fasta /home/2CI2/2CI2.fasta -in:file:frag3 /home/2CI2/aat000_03_05.200_v1_3 -in:file:frag9 /home/2CI2/aat000_09_05.200_v1_3 -database $ROSETTA_DB -abinitio:relax -relax:fast -use_filters true -psipred_ss2 /home/2CI2/t000_.psipred_ss2 -kill_hairpins /home/2CI2/t000_.psipred_ss2 -nstruct 1250 -out:file:silent 2CI2_silent.out -out:path /home/2CI2/ -mpi_tracer_to_file proc

Note that when generating SS-based predictions, the fragment files (aat000_03_05.200_v1_3 and aat000_09_05.200_v1_3)are customised beforehand, as described in the manuscript.

Since 20,000 decoys were generated per target, and we used 16 processors of a parallel machine (mpirun -np 16), each processor needed to produce 1250 decoys (-nstruct 1250). It is important to note that Rosetta’s mpi module takes care of generating random seed numbers for each of the 16 processors ensuring that the 20,000 decoys are produce through different processes.
